# Safety and effectiveness of long-term use of darbepoetin alfa in non-dialysis patients with chronic kidney disease: a post-marketing surveillance study in Japan

**DOI:** 10.1007/s10157-018-1632-9

**Published:** 2018-09-04

**Authors:** Tetsuhiro Tanaka, Masaomi Nangaku, Enyu Imai, Yoshiharu Tsubakihara, Masatoshi Kamai, Michihito Wada, Shinji Asada, Tadao Akizawa

**Affiliations:** 1 Division of Nephrology and Endocrinology, The University of Tokyo Graduate School of Medicine, Bunkyo-ku, Tokyo, Japan; 2 Internal Medicine of Nakayamadera Imai Clinic, Takarazuka, Hyogo Japan; 3 Graduate School of Health Care Sciences, Jikei Institute, Yodogawa-ku, Osaka, Japan; 4 Pharmacovigilance Department, Kyowa Hakko Kirin Co., Ltd., Chiyoda-ku, Tokyo, Japan; 5 Medical Affairs Department, Kyowa Hakko Kirin Co., Ltd., Otemachi Financial City Grand Cube, 1-9-2 Otemachi, Chiyoda-ku, Tokyo, 100-0004 Japan; 6 Showa University, Shinagawa-ku, Tokyo, Japan

**Keywords:** Non-dialysis chronic kidney disease, Darbepoetin alfa, Post-marketing surveillance, Cardiovascular-related adverse events, Composite renal endpoints

## Abstract

**Background:**

This post-marketing surveillance (PMS) study evaluated the safety and effectiveness of long-term darbepoetin alfa (darbepoetin) for the treatment of renal anemia in Japanese non-dialysis chronic kidney disease patients.

**Methods:**

Patients were treated with darbepoetin and followed up for 3 years. Adverse events (AEs), adverse drug reactions (ADRs), hemoglobin (Hb) levels, and renal function were assessed. Patients were stratified by Hb level at the time of occurrence of cardiovascular-related AEs. Statistical analyses were performed to explore factors affecting the occurrence of AEs, cardiovascular-related AEs, and composite renal endpoints.

**Results:**

In the safety analysis set (5547 patients), AEs and ADRs occurred in 44.4 and 7.1% of patients, respectively. Cardiovascular-related AEs were observed in 12.6% of the overall population. The proportion of patients who presented cardiovascular-related AEs was lower among those with a higher Hb level at the time of occurrence. In the effectiveness analysis set (5024 patients), mean Hb levels remained between 10.0 and 10.6 g/dL (Weeks 4–156). Three months after darbepoetin administration, patients with Hb ≥ 11 g/dL presented fewer composite renal endpoints than those with Hb < 11 g/dL (*p* = 0.0013), and the cumulative proportion of renal survival was higher in those with Hb ≥ 11 g/dL vs. Hb < 11 g/dL (*p* < 0.0001).

**Conclusions:**

This PMS study showed the safety and effectiveness of long-term use of darbepoetin in a large number of patients. Patients with Hb ≥ 11 g/dL presented fewer composite renal endpoints than those with Hb < 11 g/dL, without an increase in the incidence of cardiovascular-related AEs.

**Electronic supplementary material:**

The online version of this article (10.1007/s10157-018-1632-9) contains supplementary material, which is available to authorized users.

## Introduction

Renal anemia is a common complication of chronic kidney disease (CKD). The majority of patients with CKD stage 5 suffer from anemia [1]. Anemia leads to a decrease in oxygen delivery to vital organs, which is initially compensated by increased cardiac output, but may eventually result in maladaptive left ventricular hypertrophy, a well-recognized risk factor for cardiovascular (CV) disease and all-cause mortality [2, 3]. CKD patients with anemia were reported to have about twofold higher risk of CV disease than those without anemia [2, 3]. It is also suggested that progression of renal anemia not only increases the risk of CV disease, but also is an independent risk factor for the deterioration of renal function, causing the vicious cycle known as cardio-renal anemia syndrome [4].

The primary cause of renal anemia is the deficiency of endogenous erythropoietin, which is mainly produced by the kidneys [1]. Clinical studies have shown that the correction of renal anemia with erythropoiesis-stimulating agents (ESAs) might retard the progression of CKD [5, 6] and decrease left ventricular hypertrophy [7], but this is not a consistent finding as summarized in latter meta-analyses [8–12]. Moreover, lines of evidence from large-scale clinical studies in Europe and the US, which include CREATE [13], CHOIR [14] and TREAT [15], raised questions about correcting almost normal hemoglobin (Hb) levels with ESAs. Meta-analyses including these clinical studies claimed that targeting higher Hb levels in CKD increases CV risk and probably increases the risk of end-stage renal disease and death [16, 17]. However, the majority of patients recruited in these studies had diabetes or a high risk of CV disease; thus, the ideal target Hb level in the more general population of non-dialysis CKD patients, especially in Japanese patients, remains to be determined [18].

In Japan, darbepoetin alfa (darbepoetin), a long-acting ESA, became available for the treatment of renal anemia in non-dialysis CKD patients in 2010. Clinical trials of darbepoetin showed greater benefits in terms of quality of life and preserving cardiac and renal functions in Japanese non-dialysis CKD patients [19–21]. Later, a post-marketing surveillance study (Darbepoetin Alfa for Renal Anemia Management in Japan: DREAM-J, UMIN identifier: UMIN000017252) was conducted to evaluate the safety and effectiveness of long-term use of darbepoetin for the treatment of renal anemia in Japanese non-dialysis CKD patients. In addition, based on the results of clinical trials, this post-marketing surveillance study prospectively aimed at exploring the factors associated with renal and CV outcomes.

Here, we report on the adverse events (AEs) and adverse drug reactions (ADRs) of patients treated with darbepoetin in the DREAM-J study, which is thought to reflect the Japanese real-world clinical setting. Finally, we describe the relationship between treatment with darbepoetin and renal or CV outcomes in Japanese non-dialysis CKD patients.

## Materials and methods

This was a multicenter, prospective, observational post-marketing surveillance study. The protocol of the current study was approved by the Pharmaceuticals and Medical Devices Agency (PMDA). This post-marketing surveillance was part of the mandatory actions determined and reviewed by the PMDA. The study was conducted in accordance with the Japanese regulation (Ministry of Health, Labour and Welfare Ministerial Ordinance No 171) for Good Post-Marketing Study Practice (GPSP).

### Patients

Participants in the study were non-dialysis CKD patients with renal anemia who were treatment naïve for darbepoetin. Patients with planned renal replacement therapy (for dialysis or kidney transplantation) within 6 months were excluded. The target sample size was set at 3000 patients to detect at least one patient with unknown ADR occurring at a frequency of 0.1%, with a probability of ≥ 95%.

### Method

Using a central registration system, patients were registered between August 2010 and December 2011, and the survey period was from August 2010 to June 2015. The observation period continued for 3 years post-initial darbepoetin administration, and survey sheets were collected four times, at 6 months and 1, 2, and 3 years after initiation of treatment.

Discontinuation/dropout criteria included the following: discontinuation of drug administration at the physician’s discretion (excluding temporary cessation of drug administration), change to renal replacement therapy, death, and loss to follow-up due to transfer to another hospital.

Administration of darbepoetin was made according to the approved dosage and at the discretion of the treating physician at medical institutions participating in this study. Survey items were as follows: AEs including abnormal laboratory values, ADRs (i.e., AEs for which a causal relationship with darbepoetin could not be ruled out), status of darbepoetin administration (date and dosage), Hb levels, blood pressure, serum creatinine levels, urine protein (spot urine), urine creatinine, and need of transfusion. AEs and ADRs were recorded according to the International Conference on Harmonisation of Technical Requirements for Registration of Pharmaceuticals for Human Use Medical Dictionary for Regulatory Activities/Japanese version (MedDRA/J version 18.1). A CV-related AE was defined to include myocardial infarction, congestive heart failure, cerebrovascular disorders, aortic dissection, angina pectoris, chronic arteriosclerosis obliterans, and arrhythmia.

Safety evaluation involved the analysis of the occurrence of AEs and ADRs by patient characteristics. Hb levels at the time of occurrence of CV-related AEs and factors that affect the occurrence of CV-related AEs were explored.

Effectiveness evaluation included the investigation of changes in Hb levels and estimated glomerular filtration rates (eGFR) after darbepoetin administration, changes in dosage and frequency of darbepoetin administration at each point of evaluation, and factors that affect composite renal endpoints (50% reduction in eGFR, initiation of dialysis, or kidney transplantation). Patients without composite renal endpoints were defined as renal survivors.

### Statistical analysis

Proportions of patients in the safety analysis set with AEs and ADRs were analyzed by patient characteristics using Fisher’s exact test or chi-square tests. Tests were performed at a two-sided significance level of 0.05. Independent associations of potential factors with the occurrence of CV-related AEs and composite renal endpoints were assessed by Cox proportional hazards regression analysis.

The cumulative proportions of renal survival, classified by achievement or non-achievement of target Hb levels, were presented as Kaplan–Meier survival curves. For inter-group comparison, proportional hazard assumption was confirmed in each group. The log-rank test was used when the proportional hazard assumption was met (*p* < 0.05), and the generalized Wilcoxon test was used when the assumption was not met.

For multivariate Cox regression analysis using CV-related AEs as objective variables, the following variables were adjusted for potential confounders: sex, age, body mass index (BMI), history of CV disease, hypertension, hyperlipidemia, diabetes mellitus (comorbidity), previous treatment with recombinant human erythropoietin (rHuEPO), transfusion, baseline systolic blood pressure, baseline diastolic blood pressure, baseline eGFR, increased Hb level during 4 weeks of darbepoetin administration, continuous quantity of baseline urine protein, dose of darbepoetin administered per week during the study, and use of antihypertensive agents, antithrombotic agents, and lipid-lowering agents at baseline. The Cox regression analysis for CV-related AEs was performed using the safety analysis set, regardless of the darbepoetin administration period.

For multivariate Cox regression analysis using composite renal endpoints as objective variables, the following variables were selected as explanatory variables: Hb level 3 months after the start of darbepoetin administration, sex, age, BMI, previous medical history, diabetes (comorbidity), previous treatment with rHuEPO, transfusion, baseline systolic blood pressure, baseline eGFR, increased Hb level during 4 weeks of darbepoetin administration, continuous quantity of baseline urine protein, dose of darbepoetin administered per week during this study, and use of antihypertensive agents, antithrombotic agents, and lipid-lowering agents at baseline. Cox regression analysis of composite renal endpoints, in relation to Hb levels and eGFR, was performed in patients in the effectiveness analysis set who received darbepoetin for > 3 months and who did not develop any renal endpoints within 3 months.

All statistical analyses were conducted by the contract research organization Asklep Inc. (Tokyo, Japan) using SAS software, version 9.3 (SAS Institute, Inc., Cary, NC, USA).

## Results

### Patient characteristics

Between August 2010 and December 2011, 5772 patients from 966 sites were initially registered, of whom 5607 were eligible for final registration. Survey sheets were collected from 5594 patients 6 months after the start of darbepoetin administration, from 4258 patients at 1 year, 3109 patients at 2 years, and 1877 patients at 3 years. The mean observation period was 1.8 years.

**Fig. 1:**
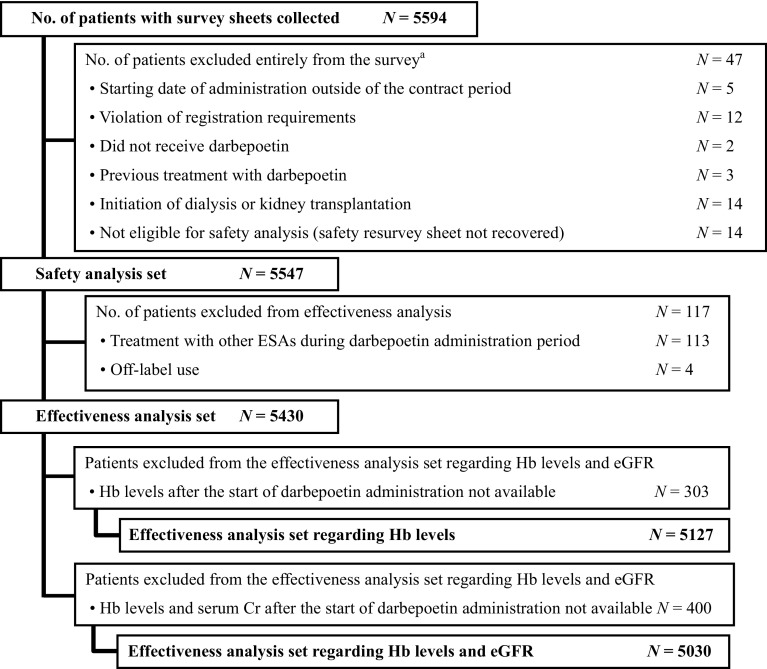
Patient disposition. ^a^If patients had more than one exclusion criterion, the patients were counted for each respective reason. *Cr* creatinine, *eGFR* estimated glomerular filtration rate, *ESAs* erythropoietin-stimulating agents, *Hb* hemoglobin

Figure 1 shows the patient disposition. A total of 47 patients were excluded from the survey, including those who initiated dialysis or underwent kidney transplantation before the start of darbepoetin administration. The remaining 5547 patients were included in the safety analysis set. Of these, the 3-year observation period was discontinued for 4309 patients; principal reasons included the initiation of dialysis (*N* = 1749) and transfer to another hospital (*N* = 1032). Of the 5547 patients in the safety analysis set, 117 patients were excluded, and thus 5430 patients were included in the main effectiveness analysis set. Patients in the effectiveness analysis with available Hb values after darbepoetin administration were further defined as the effectiveness analysis set regarding Hb levels (*N* = 5127). Those whose Hb values and serum creatinine values were available after darbepoetin administration were defined as the effectiveness analysis set regarding Hb levels and eGFR (*N* = 5030). Of the 5030 patients in the effectiveness analysis set regarding Hb levels and eGFR, these parameters were available in 5024 patients at the same evaluation point.

Patient characteristics of the safety analysis set (*N* = 5547) are presented in Table 1. Of these patients, 77.7% were elderly, 41.6% had CV disease, 5.9% had malignant tumors, and 40.5% were switched from rHuEPO. The baseline Hb level (mean ± standard deviation [SD]) was 9.5 ± 1.1 g/dL, and the baseline eGFR (mean ± SD) was 18.7 ± 10.3 mL/min/1.73 m^2^.

**Table 1:** Patient characteristics

Characteristics	Safety analysis set *N* = 5547
Sex (male)	3053 (55.0)
Age	73 ± 12
≥ 65 years	4308 (77.7)
BMI	22.7 ± 3.8
≥ 25 kg/m^2^	1014 (18.3)
Underlying disease	
Diabetic nephropathy	1818 (32.8)
Chronic glomerulonephritis	1182 (21.3)
Nephrosclerosis	1623 (29.3)
Other	924 (16.7)
Medical history	
CV disease	735 (13.3)
Malignant tumors	412 (7.4)
Comorbidity	
CV disease	2306 (41.6)
Malignant tumors	330 (5.9)
Previous treatment with rHuEPO	2244 (40.5)
Systolic blood pressure (mmHg)	134 ± 20
Hb level (g/dL)	9.5 ± 1.1
eGFR (mL/min/1.73 m^2^)	18.7 ± 10.3
Serum Cr (mg/dL)	3.1 ± 1.6

Data are shown as *n* (%) or mean ± SD

*BMI* body mass index, *Cr* creatinine, *CV* cardiovascular, *eGFR* estimated glomerular filtration rate, *Hb* hemoglobin, *rHuEPO* recombinant human erythropoietin, *SD* standard deviation

### Safety

In the safety analysis set, a total of 5735 AEs occurred in 2462 patients (44.4%), and 605 ADRs occurred in 394 patients (7.1%, Table 2). Major ADRs were hypertension (0.7%) and blood pressure increased (0.6%). Major ADRs that could not be specified in the “Precautions for Use” section of the product package (4th edition revised on December 2014) were heart failure, death, sudden death, congestive heart failure, pneumonia, and gastric cancer.

**Table 2:** Occurrence of adverse events and adverse drug reactions

	DREAM-J *N* (%)			Phase 1–3 studies on non-dialysis CKD patients in Japan^a^*N* (%)	
Safety analysis set	5547			439	
No. of AEs	5735				
No. of patients with AEs	2462 (44.4)				
No. of ADRs	605			205	
No. of patients with ADRs	394 (7.1)			135 (30.75)	
Major ADRs					
Cardiac disorders	66 (1.2)			6 (1.36)	
Cardiac failure	22 (0.4)			–	
Congestive heart failure	10 (0.2)			–	
Angina pectoris	8 (0.1)			1 (0.22)	
Nervous system disorders	49 (0.9)			16 (3.64)	
Cerebral infarction	20 (0.4)			2 (0.45)	
Vascular disorders	49 (0.9)			29 (6.60)	
Hypertension	40 (0.7)			27 (6.15)	
Benign and malignant neoplasms	37 (0.7)			–	
Gastric cancer	7 (0.1)			–	
Colon cancer	6 (0.1)			–	
Lung neoplasm malignant	6 (0.1)			–	
Blood and lymphatic system disorders	30 (0.5)			3 (0.68)	
Iron deficiency anemia	23 (0.4)			–	
Renal and urinary disorders	25 (0.5)			8 (1.82)	
General disorders and others	44 (0.8)			7 (1.59)	
Death	12 (0.2)			–	
Sudden death	10 (0.2)			–	
Skin and subcutaneous tissue disorders	31 (0.6)			8 (1.82)	
Investigations^b^	68 (1.2)			76 (17.31)	
Blood pressure increased	36 (0.6)			51 (11.61)	

ADRs are classified according to MedDRA 18.1

*ADR* adverse drug reaction, *AE* adverse event, *CKD* chronic kidney disease

^a^Quoted from an interview form in Japan [http://www.info.pmda.go.jp/go/interview/1/230124_3999425S5028_1_006_1F]

^b^Investigations include laboratory abnormalities such as blood pressure increased

Table 3 shows the occurrence of AEs and ADRs stratified by patient characteristics. CV-related AEs and malignant tumor-related AEs occurred in 12.6 and 4.1% of patients, respectively. CV-related ADRs and malignant tumor-related ADRs occurred in 2.0 and 0.6%, respectively.

**Table 3:** Adverse events and adverse drug reactions stratified by patient characteristics

Factors		No. of patients	All events (%)				CV-related events (%)				Malignant tumors (%)			
			AEs		ADRs		AEs		ADRs		AEs		ADRs	
Total		5547	2462 (44.4)		394 (7.1)		697 (12.6)		109 (2.0)		228 (4.1)		35 (0.6)	
Sex	Male	3053	1334 (43.7)		228 (7.5)		394 (12.9)		61 (2.0)		149 (4.9)		25 (0.8)	
	Female	2494	1128 (45.2)		166 (6.7)		303 (12.1)		48 (1.9)		79 (3.2)		10 (0.4)	
			n.s.		n.s.		n.s.		n.s.		*p* = 0.0014		n.s.	
Age	< 65 years	1239	439 (35.4)		53 (4.3)		87 (7.0)		12 (1.0)		33(2.7)		1 (0.1)	
	≥ 65 years	4308	2023 (47.0)		341 (7.9)		610 (14.2)		97 (2.3)		195 (4.5)		34 (0.8)	
			*p* < 0.0001		*p* < 0.0001		*p* < 0.0001		*p* = 0.0034		*p* = 0.0033		*p* = 0.0033	
Underlying disease	Diabetic nephropathy	1818	749 (41.2)		100 (5.5)		257 (14.1)		34 (1.9)		63 (3.5)		9 (0.5)	
	Chronic glomerulonephritis	1182	494 (41.8)		87 (7.4)		103 (8.7)		17 (1.4)		53 (4.5)		8 (0.7)	
	Nephrosclerosis	1623	760 (46.8)		131(8.1)		239 (14.7)		37 (2.3)		61 (3.8)		12 (0.7)	
	Others	924	459 (49.7)		76 (8.2)		98 (10.6)		21 (2.3)		51 (5.5)		6 (0.6)	
			*p* < 0.0001		*p* = 0.0104		*p* < 0.0001		n.s.		n.s.		n.s.	
History of CV disease	No	4812	2072 (43.1)		317 (6.6)		534 (11.1)		86 (1.8)		193 (4.0)		29 (0.6)	
	Yes	735	390 (53.1)		77 (10.5)		163 (22.2)		23 (3.1)		35 (4.8)		6 (0.8)	
			*p* < 0.0001		*p* = 0.0003		*p* < 0.0001		*p* = 0.0214		n.s.		n.s.	
History of malignant tumors	No	5135	2227 (43.4)		349 (6.8)		624 (12.2)		99 (1.9)		193 (3.8)		26 (0.5)	
	Yes	412	235 (57.0)		45 (10.9)		73 (17.7)		10 (2.4)		35 (8.5)		9 (2.2)	
			*p* < 0.0001		*p* = 0.0036		*p* = 0.0019		n.s.		*p* < 0.0001		*p* = 0.0008	
Comorbidity of CV disease	No	3241	1269 (39.2)		206 (6.4)		243 (7.5)		43 (1.3)		129 (4.0)		16 (0.5)	
	Yes	2306	1193 (51.7)		188 (8.2)		454 (19.7)		66 (2.9)		99 (4.3)		19 (0.8)	
			*p* < 0.0001		*p* = 0.0109		*p* < 0.0001		*p* < 0.0001		n.s		n.s.	
Comorbidity of malignant tumors	No	5217	2258 (43.3)		365 (7.0)		652 (12.5)		100 (1.9)		150 (2.9)		26 (0.5)	
	Yes	330	204 (61.8)		29 (8.8)		45 (13.6)		9 (2.7)		78 (23.6)		9 (2.7)	
			*p* < 0.0001		n.s.		n.s.		n.s.		*p* < 0.0001		*p* = 0.0001	
rHuEPO administration in past 3 months	No	3202	1373 (42.9)		217 (6.8)		384 (12.0)		55 (1.7)		119 (3.7)		16 (0.5)	
	Yes	2244	1048 (46.7)		170 (7.6)		303 (13.5)		52 (2.3)		104 (4.6)		17 (0.8)	
	Unknown, not recorded	101	41 (40.6)		7 (6.9)		10 (9.9)		2 (2.0)		5 (5.0)		2 (2.0)	
			*p* = 0.0056		n.s.		n.s.		n.s.		n.s.		n.s.	

Differences between factors were analyzed by Fisher’s exact test or the chi-square test

*ADR* adverse drug reaction, *AE* adverse event, *CV* cardiovascular, *rHuEPO* recombinant human erythropoietin, *n.s*. not significant

Table 4 shows all AEs and CV-related AEs stratified by Hb level at the time of the AE occurrence for the 5517 patients in the safety analysis set with available Hb values. The proportion of patients with CV-related AEs was lower in those with high Hb levels than in those with low Hb levels. The same tendency was observed for all AEs, AEs by MedDRA system organ class (SOC), and major CV-related AEs.

**Table 4:** All adverse events and cardiovascular-related adverse events stratified by hemoglobin level at the time of the event occurrence

	Hb level at the time of all AEs and CV-related AE occurrences									
	< 10 g/dL		≥ 10 g/dL < 11 g/dL		≥ 11 g/dL < 12 g/dL		≥ 12 g/dL < 13 g/dL		≥ 13 g/dL	
No. of all AEs	2763		1598		983		390		126	
No. of patients with all AEs (%)	1456 (26.4)		957 (17.3)		622 (11.3)		268 (4.9)		88 (1.6)	
No. of CV-related AEs	440		211		122		60		11	
No. of patients with CV-related AEs (%)	394 (7.1)		188 (3.4)		113 (2.0)		57 (1.0)		11 (0.2)	
No. of patients with CV-related AEs by type (MedDRA SOC)										
Nervous system disorders^a^	53 (1.0)		38 (0.7)		23 (0.4)		18 (0.3)		3 (0.1)	
Cardiac disorders^b^	318 (5.8)		138 (2.5)		84 (1.5)		34 (0.6)		7 (0.1)	
Vascular disorders^c^	24 (0.4)		12 (0.2)		5 (0.1)		7 (0.1)		–	
General disorders and administration site conditions^d^	12 (0.2)		6 (0.1)		5 (0.1)		1 (0.0)		1 (0.0)	
Major CV-related AEs										
Cardiac failure	151 (2.7)		59 (1.1)		27 (0.5)		11 (0.2)		2 (0.0)	
Congestive heart failure	65 (1.2)		33 (0.6)		21 (0.4)		8 (0.1)		1 (0.0)	
Cerebral infarction	20 (0.4)		27 (0.5)		5 (0.1)		11 (0.2)		1 (0.0)	

Patients in the safety analysis set with available Hb values (*n* = 5517) were subject to analysis

*AEs* adverse events, *CV* cardiovascular, *Hb* hemoglobin, *MedDRA* Medical Dictionary for Regulatory Activities, *SOC* system organ class

^a^Cerebral infarction, cerebral hemorrhage, transient ischemic attack

^b^Cardiac failure, angina pectoris, myocardial infarction

^c^Peripheral arterial occlusive disease, aortic dissection, aortic aneurysm

^d^Sudden death, cardiac death

Among the patients in the safety analysis set, 39 patients had a history of cerebrovascular disease that was found to be correlated with a history of CV disease. After excluding these patients, factors affecting the occurrence of CV-related AEs were assessed in the remaining 5508 patients by Cox regression analysis. The following factors were found to be significantly associated with the risk of CV-related AEs: advanced age (≥ 65 years), history of CV disease, presence of diabetes, transfusion, baseline eGFR < 30 mL/min/1.73 m^2^, high baseline urine protein, median darbepoetin dose administered per week of ≥ 17.1 µg, and concomitant use of antithrombotic agents (Table 5).

**Table 5:** Multivariate Cox regression analysis of cardiovascular-related adverse events

Factors		No. of patients	No. of patients with CV-related AEs (%)	HR (95% CI)	*p* value
Age (years)	< 65	1232	79 (6.4)	Ref	
	≥ 65 to < 75	1459	171 (11.7)	1.931 (1.258–2.966)	0.0026
	≥ 75	2817	404 (14.3)	2.457 (1.632–3.699)	< 0.0001
History of CV disease	No	4782	502 (10.5)	Ref	
	Yes	726	152 (20.9)	2.216 (1.656–2.966)	< 0.0001
Concurrent diabetes	No	3162	343 (10.8)	Ref	
	Yes	2346	311 (13.3)	1.561 (1.199–2.032)	0.0009
Transfusion	No	4995	547 (11.0)	Ref	
	Yes	475	105 (22.1)	1.913 (1.367–2.676)	0.0002
Baseline eGFR (mL/min/1.73 m^2^)	< 15	2227	255 (11.5)	1.849 (1.122–3.046)	0.0159
	≥ 15 to < 30	2074	279 (13.5)	1.857 (1.142–3.019)	0.0125
	≥ 30	655	61 (9.3)	Ref	
Baseline urine protein (mg/dL)	Continuous quantity	2451	260 (10.6)	1.001 (1.000–1.001)	0.0026
Dose of darbepoetin per week	< Median	2754	309 (11.2)	Ref	
	≥ Median	2754	345 (12.5)	1.361 (1.049–1.766)	0.0201
Use of antithrombotic agent	No	5018	511 (10.2)	Ref	
	Yes	490	143 (29.2)	2.377 (1.701–3.322)	< 0.0001

Patients from the safety analysis set (*n* = 5547) without a history of cerebrovascular disease (*n* = 39) were included in this multivariate analysis (*n* = 5508)

The following variables were selected as explanatory variables: sex, age, body mass index, history of CV disease, hypertension, hyperlipidemia, diabetes, previous treatment with recombinant human erythropoietin, transfusion, baseline systolic blood pressure, baseline diastolic blood pressure, baseline eGFR, increase rate of hemoglobin level during darbepoetin administration for 4 weeks, baseline urine protein (spot urine), dose of darbepoetin administered per week, antihypertensive agent, antithrombotic agent, and lipid-lowering agent. As history of CV disease and cerebrovascular disease were strongly correlated (Spearman’s rank correlation coefficient < 0.7), only history of CV disease was selected for the model

*AE* adverse event, *CI* confidence interval, *CV* cardiovascular, *eGFR* estimated glomerular filtration rate, *HR* hazard ratio

*P* values for trend (Wald chi-square test [type 3]) were *p* < 0.0001 for age and *p* = 0.0375 for baseline eGFR

The median (25, 75 percentiles) of darbepoetin dose administered per week was 17.1 (11.4, 26.5) µg

### Effectiveness

**Fig. 2:**
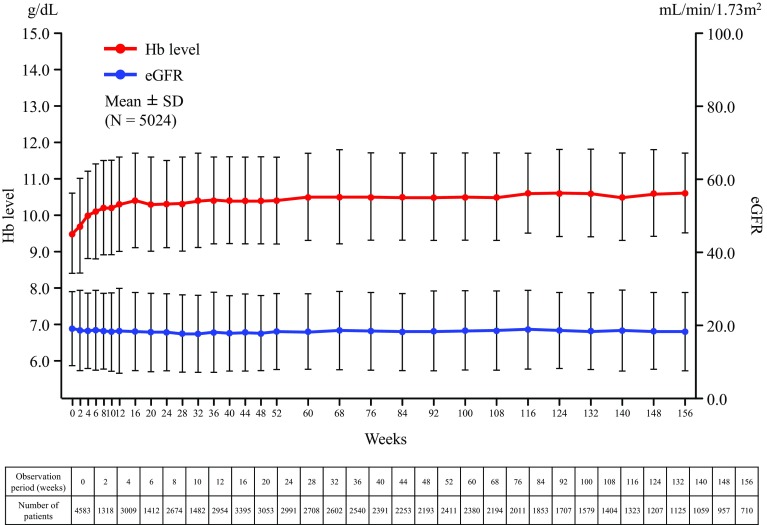
Changes in Hb levels and eGFR. *eGFR* estimated glomerular filtration rate, *Hb* hemoglobin, *SD* standard deviation

Of the 5430 patients in the effectiveness analysis set, 5024 patients with available Hb levels and eGFR at the same evaluation point were included in the analysis. The mean Hb level at baseline of 9.5 g/dL started to increase from Week 2 of darbepoetin administration and remained between 10.0 and 10.6 g/dL from Week 4 to Week 156 (Fig. 2). In the same patients, the eGFR remained almost constant throughout the observation period (18.7 ± 10.3 mL/min/1.73 m^2^ at baseline and 17.9 ± 10.8 mL/min/1.73 m^2^ at Week 156 of darbepoetin treatment; Fig. 2). The administered dose of darbepoetin (mean ± SD, µg/week) in the effectiveness analysis set regarding Hb and serum creatinine levels (*N* = 5030) was 49.9 ± 27.7 at baseline and increased gradually, reaching 76.4 ± 45.9 at Week 156. When stratified by dosing intervals, Hb levels were maintained at approximately 10.0 g/dL with each dosing interval from 3 months to 3 years of treatment (Online Resource 1).

In the effectiveness analysis set regarding Hb levels and eGFR, 4444 patients who received darbepoetin for more than 3 months and did not develop any of the renal endpoints within 3 months were followed up for 3 years for further assessment of renal endpoints (50% reduction in eGFR, initiation of dialysis, or kidney transplantation). Among these patients, 1813 patients (40.8%) developed composite renal endpoints.

The results of multivariate Cox regression analysis of factors affecting the occurrence of composite renal endpoints are shown in Table 6. Composite renal endpoints occurred in 39.5% of patients with Hb ≥ 11 g/dL at 3 months after initial darbepoetin administration compared with 44.8% of patients with Hb < 11 g/dL, demonstrating that patients with Hb ≥ 11 g/dL at 3 months after the start of darbepoetin administration had a lower risk of composite renal endpoints (hazard ratio, 0.731; 95% confidence interval, 0.604–0.884; *p* = 0.0013).

**Table 6:** Multivariate Cox regression analysis of composite renal endpoints

Factors		No. of patients	No. of patients with renal endpoints (%)	HR (95% CI)	*p* value
Hb level at 3 months after the start of darbepoetin administration	< 11 g/dL	2380	1066 (44.8)	Ref	
	≥ 11 g/dL	1093	432 (39.5)	0.731 (0.604–0.884)	0.0013
Sex	Male	2412	1071 (44.4)	Ref	
	Female	2032	742 (36.5)	0.634 (0.536–0.749)	< 0.0001
Age (years)	< 65	999	580 (58.1)	Ref	
	≥ 65 to < 75	1195	558 (46.7)	0.794 (0.652–0.967)	0.0216
	≥ 75	2250	675 (30.0)	0.669 (0.547–0.819)	< 0.0001
Diabetes	No	2567	942 (36.7)	Ref	
	Yes	1877	871 (46.4)	1.186 (1.004–1.401)	0.0444
Transfusion	No	4047	1631 (40.3)	Ref	
	Yes	364	176 (48.4)	1.424 (1.062–1.910)	0.0183
Baseline systolic blood pressure (mmHg)	< 130	1519	512 (33.7)	Ref	
	≥ 130 to < 140	851	366 (43.0)	1.468 (1.181–1.824)	0.0005
	≥ 140	1480	751 (50.7)	1.434 (1.186–1.734)	0.0002
Baseline eGFR (mL/min/1.73 m^2^)	< 15	1791	1082 (60.4)	3.227 (2.245–4.639)	< 0.0001
	15 to < 30	1729	544 (31.5)	1.344 (0.926–1.951)	0.1204
	≥ 30	538	95 (17.7)	Ref	
Baseline urine protein (mg/dL)	Continuous quantity	2033	882 (43.4)	1.001 (1.001–1.001)	< 0.0001
Dose of darbepoetin administered per week	< Median	2220	741 (33.4)	Ref	
	≥ Median	2224	1072 (48.2)	1.514 (1.282–1.790)	< 0.0001
Concurrent use of antihypertensive agent	No	650	175 (26.9)	Ref	
	Yes	3794	1638 (43.2)	1.654 (1.158–2.360)	0.0056

Patients in the effectiveness analysis set regarding Hb levels and eGFR who received darbepoetin for more than 3 months and did not develop any of the endpoints (50% reduction in eGFR, initiation of dialysis, and kidney transplantation) evaluated within 3 months of darbepoetin administration were included in this analysis (*n* = 4444)

The following variables were selected as explanatory variables: Hb level at 3 months after the start of darbepoetin administration, sex, age, body mass index, history, diabetes, previous treatment with recombinant human erythropoietin, transfusion, baseline systolic blood pressure, baseline eGFR, increase rate of Hb level during darbepoetin administration for 4 weeks, baseline urine protein (spot urine), dose of darbepoetin administered per week, antihypertensive agent, antithrombotic agent, and lipid-lowering agent

Time (days) to composite renal endpoints (either 50% reduction in eGFR, initiation of dialysis, or kidney transplantation)

*CI* confidence interval, *eGFR* estimated glomerular filtration rate, *Hb* hemoglobin, *HR* hazard ratio

*P* values for trend (Wald chi-square test [type 3]) were *p* = 0.0004 for age, *p* = 0.0002 for baseline systolic blood pressure, and *p* < 0.0001 for baseline eGFR

The median (25, 75 percentiles) of darbepoetin dose administered per week was 15.4 (10.3, 23.2) µg

Female patients or those with advanced age (≥ 65 years) had a lower risk of composite renal endpoints than did male or younger patients, while a high risk of composite renal endpoints was found in patients with diabetes, transfusion, baseline systolic blood pressure ≥ 30 mmHg, baseline eGFR < 30 mL/min/1.73 m^2^, higher baseline urine protein, median dose of darbepoetin administered per week ≥ 15.4 µg, and concomitant use of antihypertensive agents.

**Fig. 3:**
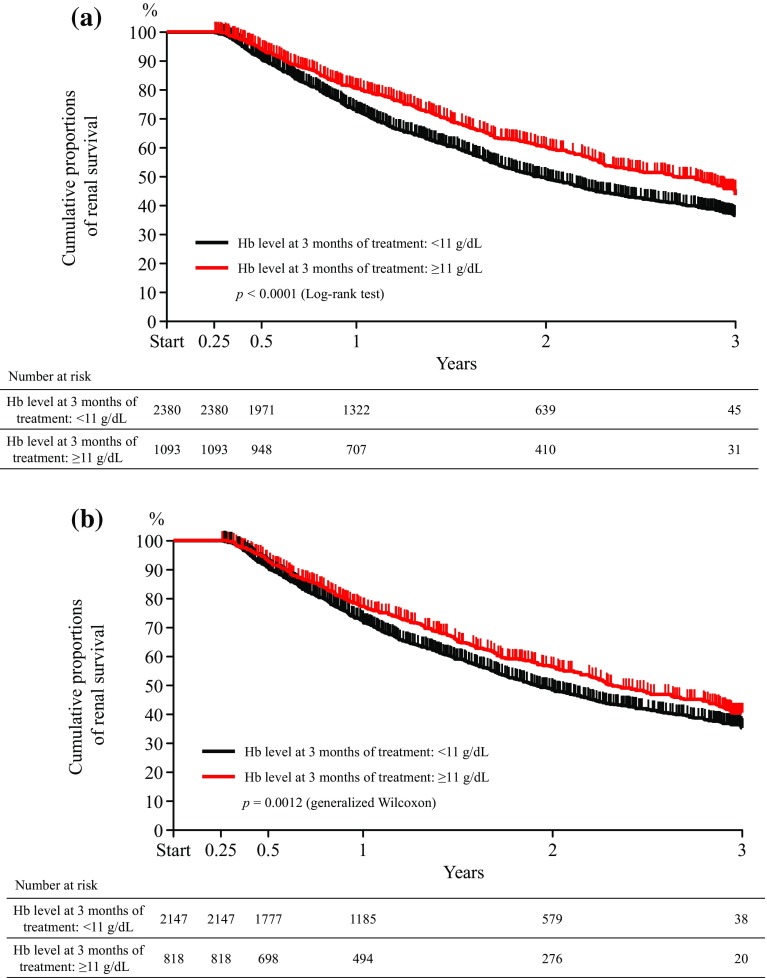
Analysis of time to first outcome on composite renal endpoints in the effectiveness analysis set for whom available Hb levels at 3 months after the start of darbepoetin administration were stratified by < 11 g/dL or ≥ 11 g/dL. **a** All patients. **b** Patients whose baseline Hb levels were < 11 g/dL. Patients who died during the course of follow-ups were excluded from the analysis. Composite renal endpoints: 50% reduction in eGFR, initiation of dialysis, or kidney transplantation. *eGFR* estimated glomerular filtration rate, *Hb* hemoglobin

Of the patients in the effectiveness analysis set regarding Hb levels and eGFR, 3473 patients with available Hb levels at 3 months after the start of darbepoetin administration were divided into two groups: those with Hb < 11 g/dL (*N* = 2380) and those with Hb ≥ 11 g/dL at 3 months after the start of darbepoetin administration (*N* = 1093). Time to occurrence of composite renal endpoints was assessed using Kaplan–Meier survival analysis (Fig. 3a). The cumulative proportion of renal survival during 3 years of darbepoetin administration was significantly higher in those with Hb ≥ 11 g/dL than those with Hb < 11 g/dL at 3 months after the start of darbepoetin administration (*p* < 0.0001, log-rank). A sensitivity analysis, which was performed for patients with Hb < 11 g/dL at baseline, showed a similar result that the cumulative proportion of renal survival was higher in those with Hb ≥ 11 g/dL than those with Hb < 11 g/dL at 3 months after the start of darbepoetin administration (*p* = 0.0012, generalized Wilcoxon test, Fig. 3b).

Multivariate Cox regression analysis and Kaplan–Meier survival analysis of time to event provided similar results even after death was included in composite renal endpoints (Online Resources 2 and 3a, b).

## Discussion

This paper reports on the results of the DREAM-J surveillance study conducted to explore the factors that affect the occurrence of ADRs, safety, and effectiveness of long-term use of darbepoetin in non-dialysis CKD patients with renal anemia. In this study, ADRs occurred in 7.1% of patients. The ADR rate in this study was not higher than that in phase 1–3 studies on non-dialysis CKD patients in Japan [19–21]. Furthermore, major ADRs that could not be specified in the “Precautions for Use” section of the product package (4th edition revised on December 2014) were heart failure, death, sudden death, congestive heart failure, pneumonia, and gastric cancer. These ADRs had been demonstrated in clinical studies in other countries [15, 22]. According to these safety results, no new safety concerns were identified in the present study.

The incidence rate of ADRs in the present study (7.1%) was lower than that reported in pre-approval Japanese clinical trials (30.75%) [19–21]. Regarding ADRs classified by SOC, cardiac disorders occurred in 1.2% of patients, which was similar to the occurrence of cardiac disorders reported in the pre-approval Japanese clinical studies (1.36%) [19–21]. Although no malignant tumors occurred in the pre-approval Japanese clinical studies, they occurred in 0.6% of the patients in this study. Data from the Japan Cancer Surveillance Research Group (Cancer Information Service, National Cancer Center, Japan) have shown that crude incidence rates of cancer in Japan were 0.76% for men and 0.54% for women in 2009 [23], suggesting that the incidence rate of 0.6% in this study (average, 1.8 years; maximum, 3 years) is roughly comparable to that of the general population in Japan. Therefore, the discrepancy between this study and the clinical studies may be attributable to the fact that patients with a history of malignant tumor were not excluded from this study and that the observation period was rather long. Finally, hypertension, increase in blood pressure, and other ADRs that occur relatively frequently developed at similar frequencies as reported in the pre-approval Japanese clinical studies and overseas large-scale clinical studies [13–15].

In the DREAM-J surveillance study, the incidence of CV-related AEs did not tend to increase in patients with higher Hb levels. In large-scale clinical studies outside Japan, concern was raised that treatment of non-dialysis CKD patients with renal anemia using ESA with the aim of normalizing Hb levels may be associated with increased risk of CV disease. In the CHOIR study (target Hb, 13.5 g/dL) [14], there were increased incidences of myocardial infarction, hospitalization due to heart failure, and stroke in patients with higher Hb levels; whereas, in the TREAT study (target Hb, ≥ 13.0 g/dL) [15], an increased incidence of cerebral infarction in patients with higher Hb levels was noted. However, in randomized controlled studies of non-dialysis CKD patients with renal anemia conducted in Japan, no difference was found between the darbepoetin group (target Hb, 11.0–13.0 g/dL) and the rHuEPO group (target Hb, 9.0–11.0 g/dL) in terms of safety, including risk for CV events. However, there was a significant decrease in the left ventricular mass index in the darbepoetin group [19, 20]. The present study revealed no association between Hb levels and the risk of CV-related AEs, including cerebral infarction, cardiac failure, and congestive heart failure, thus supporting the results obtained in Japanese randomized controlled trials [19–21]. The trends for all AEs and CV-related AEs by Hb values were similar. Taken together, these results suggest that there is no apparent risk for CV events under clinical conditions where darbepoetin treatment is prescribed in accordance with Japanese guidelines for the management of anemia (2008) [24].

This study demonstrated that a decrease in composite renal endpoints is associated with the achievement of target Hb levels being maintained by darbepoetin. Kaplan–Meier survival analysis of time to occurrence of composite renal endpoints revealed a significantly lower incidence of events in patients who achieved an Hb ≥ 11 g/dL at 3 months after the start of darbepoetin administration. Moreover, multivariate Cox regression analysis identified the maintenance of Hb ≥ 11 g/dL 3 months after the start of darbepoetin administration as a significant factor for decreased risk of composite renal endpoints. These results are consistent with those obtained with rHuEPO in previous studies [5, 6, 25]. These results also support the findings of a study in which darbepoetin treatment was given for 3 years while Hb levels were maintained at a target of 11.0–13.0 g/dL, and a 29% risk reduction for composite renal endpoints was found [21].

However, reports of studies outside Japan suggest that there is no relationship between ESA treatment and preservation of renal function. In the CREATE study [13], dialysis was required in a significantly higher proportion of patients with high Hb levels, while no difference was seen between high and low Hb groups in terms of reduction in eGFR. In the CREATE study, the target Hb value in the high Hb group was as high as 13.0–15.0 g/dL, and hypertension (defined as a systolic blood pressure of ≥ 160 mmHg) was reported as an AE in more patients in the high Hb group (20% of patients in the low Hb group vs. 30% in the high Hb group). ESA treatment has been reported to exert kidney protection independent from its correction of anemia and result in the improvement of renal hemodynamics, as shown by the induction of an anti-oxidative enzyme involved in endothelial function [26], and an anti-apoptotic effect in glomerular epithelial cells [27]. In this study, increased systolic blood pressure was shown to be a significant risk factor for composite renal endpoints. Given that ESAs often cause increased blood pressure, appropriate management of this is likely to contribute to preventing composite renal endpoints.

In the multivariate Cox models, patients who received higher doses of darbepoetin had increased risks of composite renal endpoints and CV-related AEs. It is possible that this is related to ESA hyporesponsiveness. ESA hyporesponsiveness caused various clinical conditions including bleeding/blood loss, hematopoietic disorder (infection, inflammation, and malignant tumor), deficiency of elements required for erythropoiesis (iron, folic acid, and vitamin B12 deficiency), and anti-erythropoietin antibody [28]. In the secondary analysis of the TREAT study, patients with ESA hyporesponsiveness had significantly higher rates of death and cardiovascular events [29]. In that study, there were no apparent increases in CV-related AEs at higher Hb levels. Therefore, ESA hyporesponsiveness may increase renal and CV risk in patients who receive higher doses of darbepoetin. In the present study, a lower incidence of CV-related AEs was observed in patients with Hb ≥ 11 g/dL than in patients with Hb < 11 g/dL. The latest guidelines at the start of the current study (2008) [24] recommended an Hb level of 11.0–13.0 g/dL. In the most recent guideline (2015), the same target range has been adopted [28]. The results of the current study validate the lower range of the target Hb level (11.0 g/dL) recommended by the Japanese guidelines [24, 28]. The examination of CV-related AEs by patient characteristics revealed that an increase in CV-related AEs was found in patients with a history of CV disease and of CV-related comorbidity. Multivariate Cox regression analysis identified advanced age, diabetes, transfusion, low eGFR values, high urine protein, and use of antithrombotic agents as independent risk factors for increased CV-related AEs. Careful consideration should, therefore, be given to CV-related AEs when darbepoetin is administered to such patients.

### Limitations

This post-marketing surveillance is an observational study conducted under the rules of GPSP; as such, it is expected to show reporting bias concerning AEs compared with clinical trials (intervention studies) conducted under GCP guidelines. There was no control group to allow a comparison of safety and effectiveness and no comparison of AEs and prognosis. Additionally, data from patients who withdrew from the study or switched to renal replacement therapies were not provided. As part of the design of this study, AEs were collected according to spontaneous reports, and it appears that the incidence of AEs was lower than in clinical studies [19–21]. Although caution should be applied when comparing the incidence in this study and clinical studies, it is thought that this result may reflect the underlying medical condition as this drug was used for a wide range of patients. In this study, we could not evaluate multivariate Cox models adjusted for iron status, because we did not obtain laboratory results on iron status, or information regarding iron intake. Furthermore, in this study, the prescription of darbepoetin is based solely on the clinical judgment of the nephrologists in charge of the patients. Therefore, the influence of facility or physician bias cannot be excluded. Despite these limitations, this is a large-scale, prospective study conducted under clinical conditions in Japan and reflects the *status quo* of medical practice in Japan. Further large-scale, prospective studies are required to verify the effects of the correction of Hb levels using darbepoetin on CV-related events and composite renal endpoints.

## Conclusions

The DREAM-J surveillance study demonstrated the safety profile of darbepoetin under clinical conditions in Japan. The study results did not suggest an increase in the risk of CV-related AEs under high Hb levels. Additionally, our findings illustrate that darbepoetin treatment with a target Hb level of ≥ 11 g/dL, as recommended by the guidelines, is associated with a lower occurrence of composite renal endpoints at an early stage of CKD. Results from this surveillance study provide further insights into CKD in Japan.

## Electronic supplementary material

Below is the link to the electronic supplementary material.

Supplementary material 1 (PDF 84 KB)

Supplementary material 2 (PDF 142 KB)
