# Supplementary material for: Safety and effectiveness of long-term use of darbepoetin alfa in non-dialysis patients with chronic kidney disease: a post-marketing surveillance study in Japan
Source: Clin Exp Nephrol. 2018 Sep 4;23(2):231–43. doi: 10.1007/s10157-018-1632-9 (PMC6510805; doi:10.1007/s10157-018-1632-9)
Supplement: Supplementary file 1 — Supplementary material 1 (PDF 84 KB) [file 10157_2018_1632_MOESM1_ESM.pdf]

Online Resource 3. Analysis of time to first outcome of composite renal endpoints, including death, in the patients in the effectiveness analysis set with available Hb levels at 3 months after the start of darbepoetin administration were stratified by Hb <11 g/dL or >=11 g/dL.

(a) All patients.

(b) Patients whose baseline Hb levels were <11 g/dL. Composite renal endpoints: 50% reduction in eGFR, initiation of dialysis, kidney transplantation, or death.

Abbreviations: eGFR, estimated glomerular filtration rate; Hb, hemoglobin

Online Resource 3a.

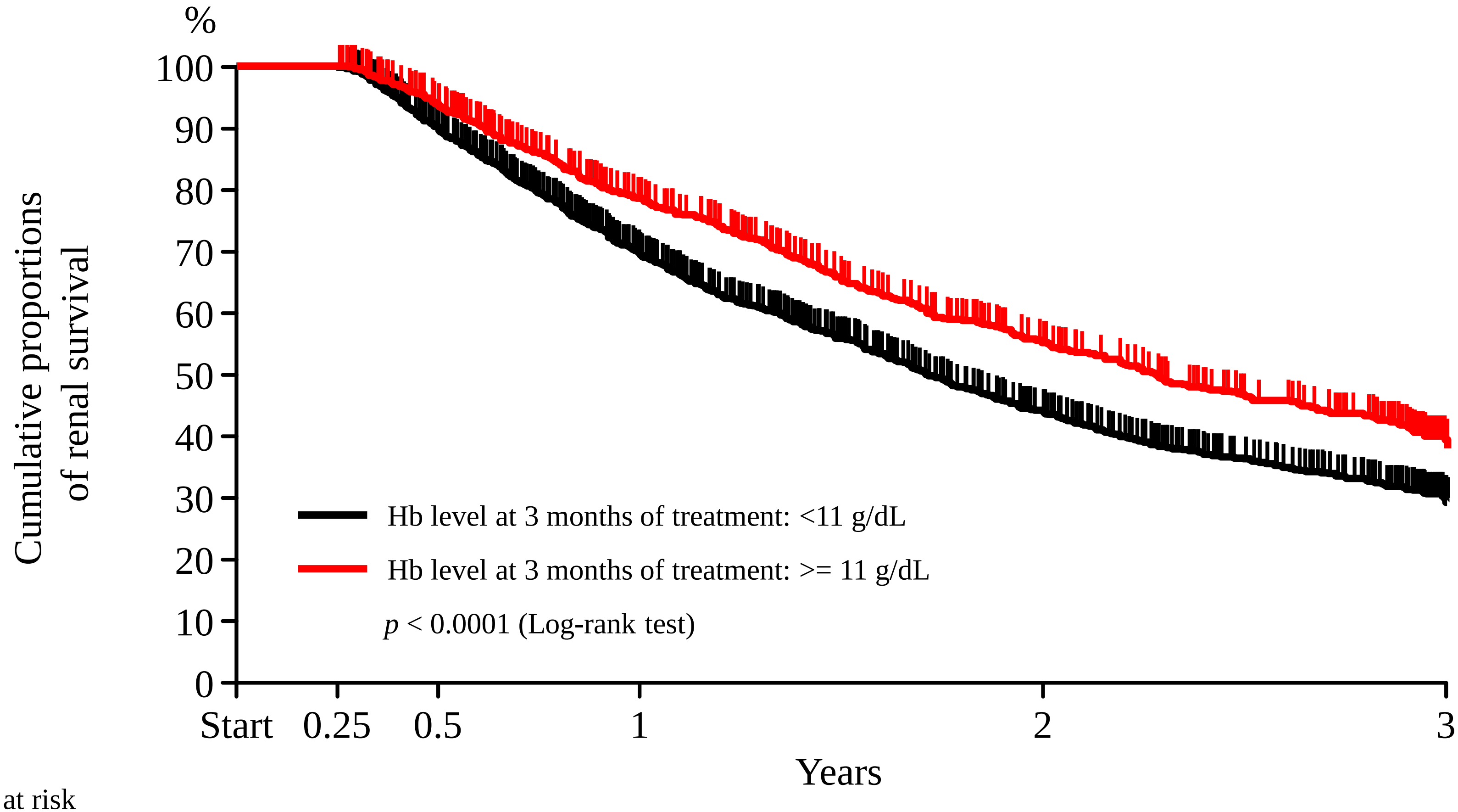

Number at risk

	Start	0.25	0.5	1	2	3
Hb level at 3 months of treatment: <11 g/dL	2380	2380	1988	1331	653	48
Hb level at 3 months of treatment: >=11 g/dL	1093	1093	950	711	413	31

Online Resource 3b.

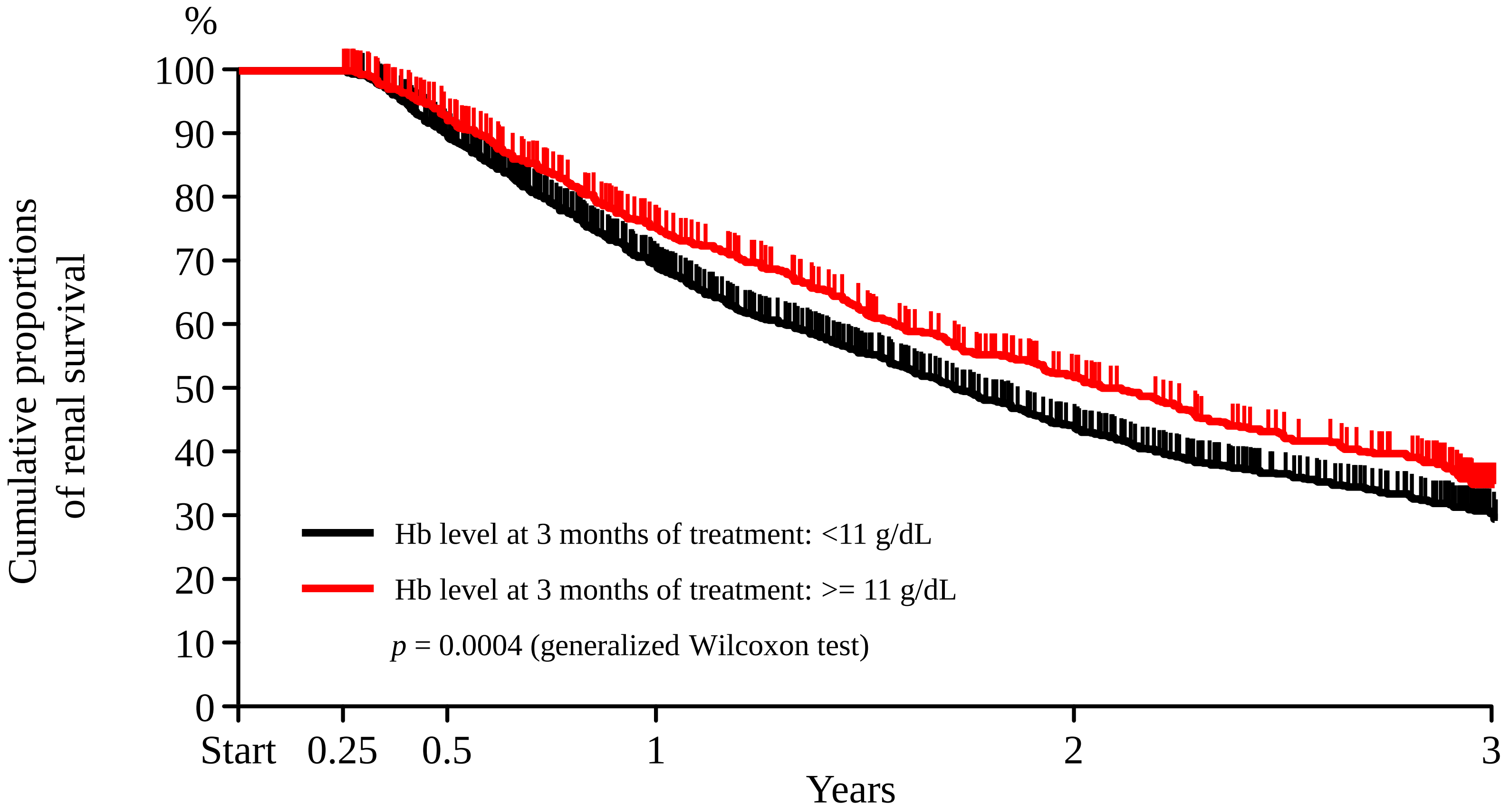

Number at risk

	Start	0.25	0.5	1	2	3
Hb level at 3 months of treatment: <11 g/dL	2147	2147	1793	1194	588	41
Hb level at 3 months of treatment: >=11 g/dL	818	818	699	498	278	20
