# Supplementary material for: Safety and effectiveness of long-term use of darbepoetin alfa in non-dialysis patients with chronic kidney disease: a post-marketing surveillance study in Japan
Source: Clin Exp Nephrol. 2018 Sep 4;23(2):231–43. doi: 10.1007/s10157-018-1632-9 (PMC6510805; doi:10.1007/s10157-018-1632-9)
Supplement: Supplementary file 2 — Supplementary material 2 (PDF 142 KB) [file 10157_2018_1632_MOESM2_ESM.pdf]

Supplementary Materials

Online Resource 1. Changes in Hb levels and doses of darbepoetin administration stratified by dosing intervals

	Dosing intervals	Observation period (years)					
		Baseline	0.25	0.5	1	2	3
Hb level (g/dL)	<2 weeks	8.9 ± 1.2 (294)	10.1 ± 1.6 (83)	9.9 ± 1.5 (61)	10.2 ± 1.2 (51)	10.3 ± 1.1 (24)	9.8 ± 1.0 (15)
	>=2 weeks, <3 weeks	9.3 ± 1.2 (1,235)	10.2 ± 1.5 (672)	10.3 ± 1.4 (485)	10.2 ± 1.4 (334)	10.5 ± 1.5 (134)	10.4 ± 1.3 (98)
	>=3 weeks, <5 weeks	9.6 ± 1.1 (1,900)	10.3 ± 1.3 (1,707)	10.3 ± 1.2 (1,485)	10.5 ± 1.2 (1,098)	10.5 ± 1.2 (655)	10.7 ± 1.1 (376)
	>=5 weeks	9.7 ± 1.0 (1,272)	10.4 ± 1.2 (1,655)	10.4 ± 1.2 (1,526)	10.5 ± 1.2 (1,116)	10.6 ± 1.2 (666)	10.5 ± 1.1 (242)
Dose of darbepoetin administration (μg per week)	<2 weeks	54.6 ± 31.8 (334)	65.2 ± 34.8 (87)	78.2 ± 48.9 (68)	76.3 ± 60.0 (54)	72.3 ± 38.4 (27)	92.9 ± 52.9 (35)
	>=2 weeks, <3 weeks	39.3 ± 18.2 (1,374)	46.8 ± 24.7 (700)	50.2 ± 27.3 (519)	54.0 ± 30.6 (357)	57.5 ± 32.9 (154)	62.8 ± 40.0 (157)
	>=3 weeks, <5 weeks	27.3 ± 14.9 (2,069)	30.4 ± 16.4 (1,763)	33.6 ± 19.4 (1,549)	35.3 ± 20.5 (1,154)	37.1 ± 22.4 (693)	38.4 ± 22.5 (585)
	>=5 weeks	17.5 ± 10.6 (1,350)	19.7 ± 12.9 (1,755)	19.4 ± 13.5 (1,635)	21.3 ± 15.8 (1,201)	23.1 ± 16.7 (744)	25.9 ± 16.6 (422)

Data are shown as mean ± standard deviation and number of patients in parentheses. Abbreviation: Hb, hemoglobin

Online Resource 2. Multivariate Cox Regression analysis in composite renal endpoints including death

Factors		No. of patients	No. of patients with renal endpoints (%)		HR (95% CI)		*p* value
Hb level at 3 months after the start of darbepoetin administration	<11 g/dL	2,380	1,255	(52.7)	Ref		
	>=11 g/dL	1,093	503	(46.0)	0.745	(0.623–0.890)	0.0012
Sex	Male	2,412	1,262	(52.3)	Ref		
	Female	2,032	882	(43.4)	0.622	(0.531–0.729)	<0.0001
Age (years)	<65	999	597	(59.8)	Ref		
	>=65 to <75	1,195	603	(50.5)	0.817	(0.675–0.989)	0.0383
	>=75	2,250	944	(42.0)	0.775	(0.641–0.936)	0.0082
Diabetes	No	2,567	1,150	(44.8)	Ref		
	Yes	1,877	994	(53.0)	1.180	(1.009–1.380)	0.0378
Transfusion	No	4,047	1,893	(46.8)	Ref		
	Yes	364	243	(66.8)	1.614	(1.248–2.088)	0.0003

Baseline systolic blood pressure (mmHg)	<130	1,519	640	(42.1)	Ref		
	>=130 to <140	851	416	(48.9)	1.389	(1.134–1.701)	0.0015
	>=140	1,480	843	(57.0)	1.326	(1.110–1.583)	0.0019
Baseline eGFR (mL/min/1.73 m^2)	<15	1,791	1,172	(65.4)	2.906	(2.086–4.048)	<0.0001
	>=15 to <30	1,729	698	(40.4)	1.346	(0.960–1.887)	0.0850
	>=30	538	141	(26.2)	Ref		
Baseline urine protein (mg/dL)	continuous quantity	2,033	1,003	(49.3)	1.001	(1.001–1.001)	<0.0001
Dose of darbepoetin administered per week	<Median	2,220	882	(39.7)	Ref		
	>=Median	2,224	1,262	(56.7)	1.505	(1.287–1.761)	<0.0001
Concurrent use of antihypertensive agent	No	650	260	(40.0)	Ref		
	Yes	3,794	1,884	(49.7)	1.425	(1.053–1.929)	0.0218

Patients in the effectiveness analysis set regarding Hb levels and eGFR who received darbepoetin for more than 3 months and did not develop any of the endpoints evaluated within 3 months of darbepoetin administration were included in this analysis (n = 4,444).

The following variables were selected as explanatory variables: Hb level at baseline, sex, age, body mass index, history, diabetes, previous treatment with recombinant human erythropoietin, transfusion, baseline systolic blood pressure, baseline diastolic blood pressure, baseline eGFR, increase rate of Hb level during darbepoetin administration for 4 weeks, baseline urine protein (spot urine), dose of darbepoetin administered per week, antihypertensive agent, antithrombotic agent, and lipid-lowering agent.

Time (days) to composite renal endpoints (either 50% reduction in eGFR, initiation of dialysis, kidney transplantation, or death).

P values for the trend (Wald chi-square test [type 3]) were $p = 0.0213$ for age, $p = 0.0012$ for baseline systolic blood pressure, and $p < 0.0001$ for baseline eGFR.

The median (25th, 75th percentiles) of darbepoetin dose administered per week was 15.4 (10.3, 23.2) μg.

Abbreviations: Hb, hemoglobin; HR, hazard ratio; CI, confidence interval; eGFR, estimated glomerular filtration rate
